# Genotypic Differences in Potato Yield and Starch Content Response to Potassium Fertilization Under Drought Stress

**DOI:** 10.3390/plants15152386

**Published:** 2026-08-04

**Authors:** Yuying Sang, Dong Wang, Yikun Li, Handa Zhang, Mingshou Fan, Ziyi Zhang

**Affiliations:** 1 Inner Mongolia Key Laboratory of Plants Adversity Adaptation and Genetic Improvement in Cold and Arid Regions, College of Life Sciences, Inner Mongolia Agricultural University, Hohhot 010018, China; 18304896891@163.com (Y.S.); wd0127@126.com (D.W.); 17658155562@163.com (Y.L.); 2 Inner Mongolia Academy of Agricultural and Animal Husbandry Sciences, Hohhot 010010, China; zhd13514717954@163.com; 3 College of Agriculture, Inner Mongolia Agricultural University, Hohhot 010018, China; fmswh@126.com

**Keywords:** potato, drought stress, potassium fertilization, tuber yield, starch content, genotypic variation

## Abstract

Drought stress constrains potato (*Solanum tuberosum* L.) production, and potassium (K) fertilization may alleviate it, but genotype-dependent responses remain unclear. Six cultivars (‘V7’, ‘DXY’, ‘JZS-3’, ‘SY-2’, ‘JZS-12’, ‘ZJ-7’) were grown under four treatments: low K with normal irrigation (T1) or drought (T2), and K application with normal irrigation (T3) or drought (T4). Dry matter in roots, stems, leaves, and tubers was measured at 50–110 days after planting, starch content at 70–110 days, and final yield recorded. Drought (T2) reduced yield by 28.6–52.3% versus T1; K under drought (T4) recovered yield by 12.8–41.5%. Starch decreased by 18.5–35.2% under drought but was restored to 82.6–94.3% of T1 levels with K. SY-2 showed the highest biomass and yield across treatments, while V7 was most affected. Based on yield responses, cultivars fell into four categories: K-efficient/drought-tolerant (SY-2), K-responsive/drought-tolerant (DXY), K-non-responsive/drought-tolerant (JZS-12), and K-inefficient/drought-sensitive (V7). K application mitigates drought-induced losses, but benefits are genotype-dependent. This classification supports genotype-specific K recommendations in water-limited systems, and SY-2 is a valuable genetic resource for breeding for K-use efficiency and drought tolerance. Physiological assessments of leaf relative water content and stomatal conductance confirmed that drought treatment effectively induced water stress and revealed that genotype-specific stomatal regulation underpinned the differential yield responses to K fertilization under drought.

## 1. Introduction

Potato (*Solanum tuberosum* L.) is the world’s largest non-cereal food crop, with a cultivation area of approximately 4.81 million hectares in China as of 2018 [1]. Its high yield potential, nutritional value, and extensive industrial applications make it a critical component of global food security [2]. However, potato production is increasingly threatened by water scarcity, particularly in arid and semi-arid regions such as Inner Mongolia, where rainfall is concentrated in July–August and annual precipitation averages only 463 mm [3]. Drought stress has been identified as a primary abiotic constraint limiting potato yield, with reported reductions of 20–50% depending on stress intensity and genotype [4].

Potassium is one of the three essential macronutrients for crop production, and potato is recognized as a typical “K-loving” crop with exceptionally high K demand [5]. The critical roles of K in potato production are well established: K promotes photosynthetic assimilate translocation from source to sink organs, enhances sucrose-to-starch conversion through activation of key enzymes such as sucrose synthase (SuSy) and ADP-glucose pyrophosphorylase (AGPase), and improves tuber quality attributes including starch content and reducing sugar balance [6,7,8,8,9]. Field studies have demonstrated that optimal K fertilization can increase potato tuber yield by 13.0–45.0%, and in some cases by over 120% in severely K-deficient soils [10,11].

Despite the recognized importance of K in potato production, two critical knowledge gaps remain. First, most studies have focused on single cultivars or limited genotypes, making it difficult to assess whether K-induced drought mitigation is a general phenomenon or a genotype-specific response [12]. Second, the relationship between a cultivar’s inherent K-use efficiency and its drought tolerance—and whether these two traits are genetically coupled—has not been systematically evaluated across diverse potato germplasm. Understanding this relationship is essential for both breeding programs targeting stress-tolerant varieties and for developing genotype-specific K fertilizer recommendations [13,14].

This study was therefore designed to (1) quantify the effects of K application on dry matter accumulation, tuber yield, and starch content across six potato cultivars with contrasting genetic backgrounds under both well-watered and drought-stressed conditions; (2) classify cultivars based on their combined K-use efficiency and drought tolerance; and (3) identify genotypes that exhibit both high K-use efficiency and strong drought tolerance as candidate genetic resources for future breeding and precision K management strategies.

## 2. Results

### 2.1. Dry Matter Accumulation in Roots, Stems, Leaves, and Tubers

#### 2.1.1. Root Dry Matter

**Figure 1:**
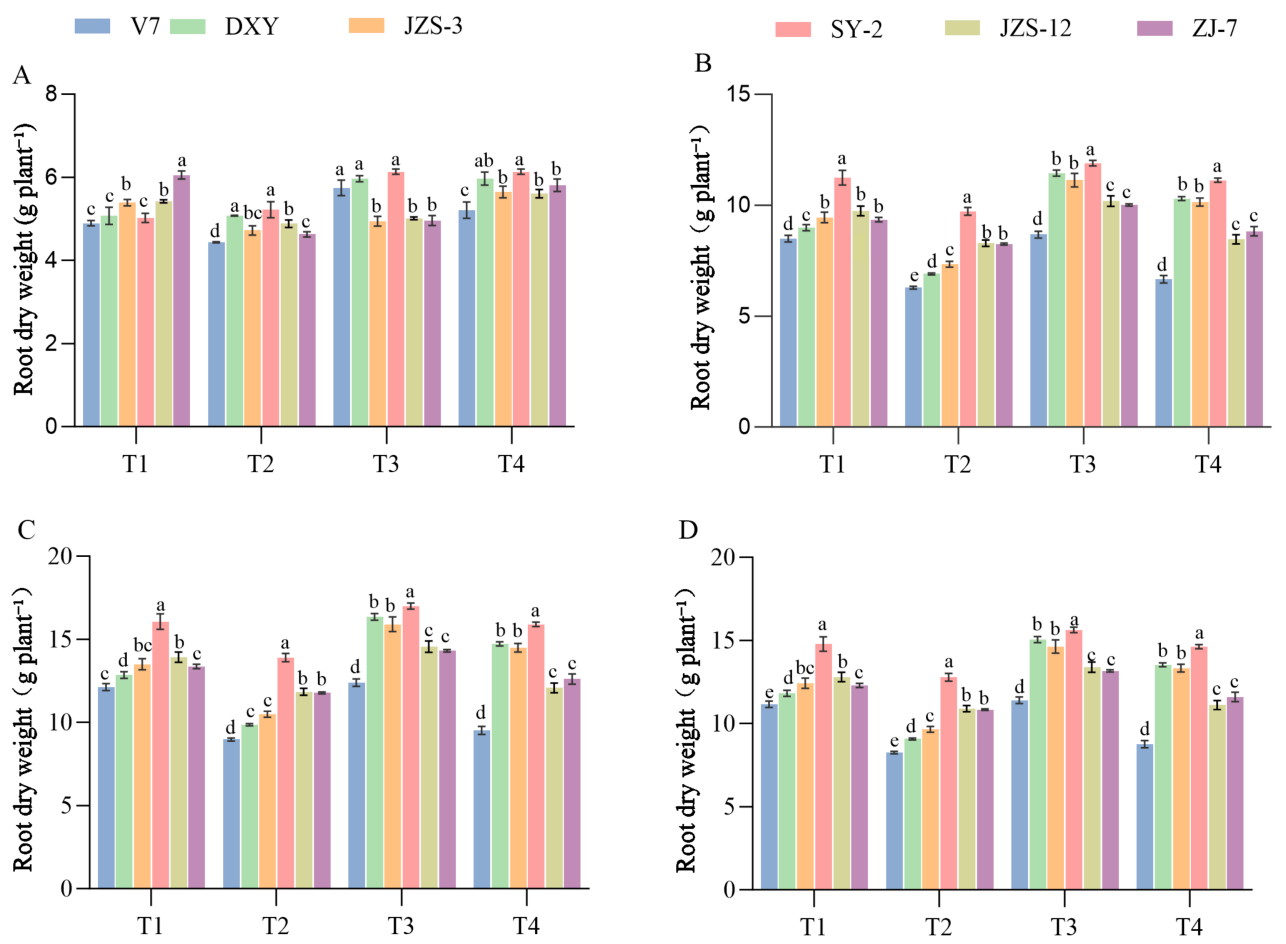
Dynamic changes in root dry matter accumulation of six potato cultivars under different treatments across the growing season (50–110 days after planting, DAP). (**A**–**D**) represent measurement time points at 50, 70, 90, and 110 DAP, respectively. The x-axis for each panel represents the six cultivars (V7, DXY, JZS-3, SY-2, JZS-12, ZJ-7), and the y-axis represents root dry weight (g plant^−1^). Four treatments are shown: T1 (normal irrigation, no K), T2 (drought stress, no K), T3 (normal irrigation + K application, 300 kg K_2_O ha^−1^), and T4 (drought stress + K application, 300 kg K_2_O ha^−1^). Data are presented as means ± SE (*n* = 3). Different lowercase letters above the bars within each cultivar indicate significant differences among treatments at *p* < 0.05 based on Fisher’s LSD test after two-way ANOVA. Root dry matter followed a unimodal pattern across all cultivars, peaking at 90 DAP and declining slightly thereafter. SY-2 consistently exhibited the highest root dry weight across treatments, whereas V7 showed the lowest, particularly under drought (T2 and T4).

Root dry matter accumulation followed a unimodal pattern across the growing season, peaking at 90 DAP and declining slightly thereafter (Figure 1). Drought stress (T2) significantly inhibited root growth across all cultivars at all time points. Cultivar SY-2 consistently exhibited the highest root dry weight across all four treatments and four time points. In contrast, V7 showed the lowest root dry weight, particularly under T2, indicating its high sensitivity to water deficit. K application significantly alleviated drought-induced root growth inhibition, with the alleviation effect being more pronounced at 90 and 110 DAP (mid-to-late season). Among cultivars, DXY and JZS-3 showed the strongest positive responses to K application, while JZS-12 and ZJ-7 displayed moderate drought tolerance with relatively stable root performance under T2.

#### 2.1.2. Stem Dry Matter

**Figure 2:**
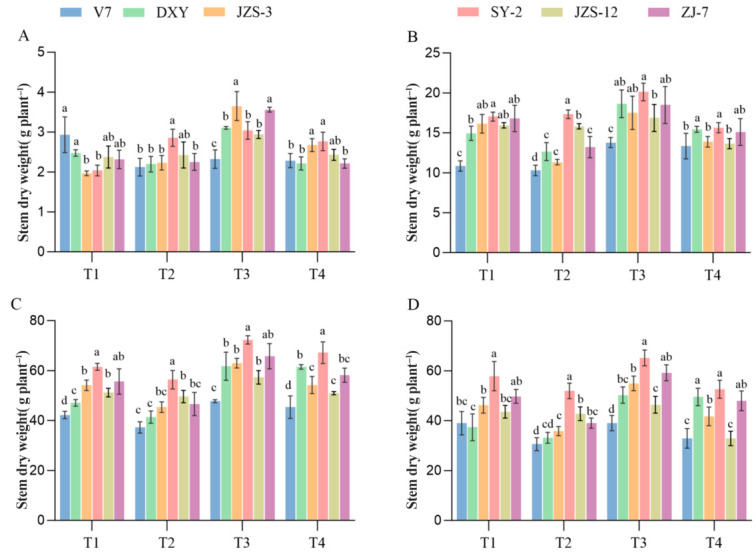
Effects of drought and potassium application on stem dry weight of six potato cultivars (50–110 DAP). (**A**–**D**) correspond to 50, 70, 90, and 110 DAP, respectively. The x-axis shows the six cultivars (V7, DXY, JZS-3, SY-2, JZS-12, ZJ-7), and the y-axis indicates stem dry weight (g plant^−1^). Treatments are T1 (normal irrigation, no K), T2 (drought, no K), T3 (normal irrigation + K), and T4 (drought + K), with K applied at 300 kg K_2_O ha^−1^. Values are means ± SE (*n* = 3). Bars with different letters within each cultivar–time combination are significantly different at *p* < 0.05 (LSD test). Stem dry matter increased from 50 to 90 DAP and then stabilized. At 90 DAP, T2 reduced stem dry weight by 38.0% on average compared with T1, while T4 partially alleviated this reduction (14.0–28.0% increase over T2). SY-2 had the highest stem dry weight across all treatments, and V7 the lowest (as low as 28.4 g plant^−1^). Genotypic differences in K responsiveness were evident: DXY responded strongly (+28.0%, T4 vs. T2), whereas JZS-12 showed a modest increase (+14.0%).

Stem dry matter increased from 50 to 90 DAP and then stabilized (Figure 2). T2 reduced stem dry weight by an average of 38.0% at 90 DAP compared with T1. K application (T3 and T4) partially restored stem growth, with T4-treated plants showing 14.0–28.0% higher stem dry weight compared with T2. SY-2 exhibited the highest stem dry weight across all treatments and time points, while V7 consistently showed the lowest values (as low as 28.4 g/plant). DXY showed strong K responsiveness (28.0% increase in T4 vs. T2), whereas JZS-12 showed only modest improvement (14.0% increase), suggesting genotype-specific K responsiveness.

#### 2.1.3. Leaf Dry Matter

**Figure 3:**
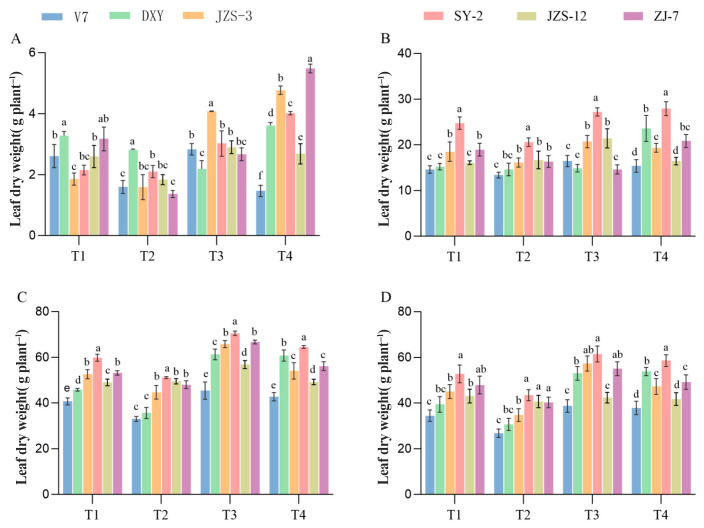
Leaf dry matter accumulation of six potato cultivars under different treatments (50–110 DAP). (**A**–**D**) represent sampling dates at 50, 70, 90, and 110 DAP, respectively. The x-axis lists the six cultivars (V7, DXY, JZS-3, SY-2, JZS-12, ZJ-7), and the y-axis shows leaf dry weight (g plant^−1^). Treatments are T1 (normal irrigation, no K), T2 (drought, no K), T3 (normal irrigation + K), and T4 (drought + K). K was applied at 300 kg K_2_O ha^−1^. Data are means ± SE (*n* = 3). Different letters above bars indicate significant differences among treatments within each cultivar at *p* < 0.05 (LSD test). Drought (T2) reduced leaf dry weight by 31.5% on average compared with T1; K application (T4) recovered 42.3% of the drought-induced loss. SY-2 maintained the highest leaf dry weight across treatments, while V7 had the lowest. DXY showed the strongest K response (+46.5% under T3 vs. T1), whereas JZS-12 exhibited the smallest drought reduction (−16.4% under T2 vs. T1), indicating strong inherent drought tolerance.

Leaf dry matter accumulation patterns (Figure 3) were similar to those of stems. T2 reduced leaf dry weight by an average of 31.5% across cultivars. T4 significantly mitigated this reduction, with an average alleviation rate of 42.3%. SY-2 maintained the highest leaf dry weight across all treatments and time points. DXY showed the strongest K response (46.5% increase under T3 vs. T1). V7 consistently exhibited the lowest leaf dry weight across all treatments. JZS-12 showed relatively small drought-induced reductions (only 16.4% reduction under T2 vs. T1), indicating excellent inherent drought tolerance of its leaf system.

#### 2.1.4. Tuber Dry Matter

**Figure 4:**
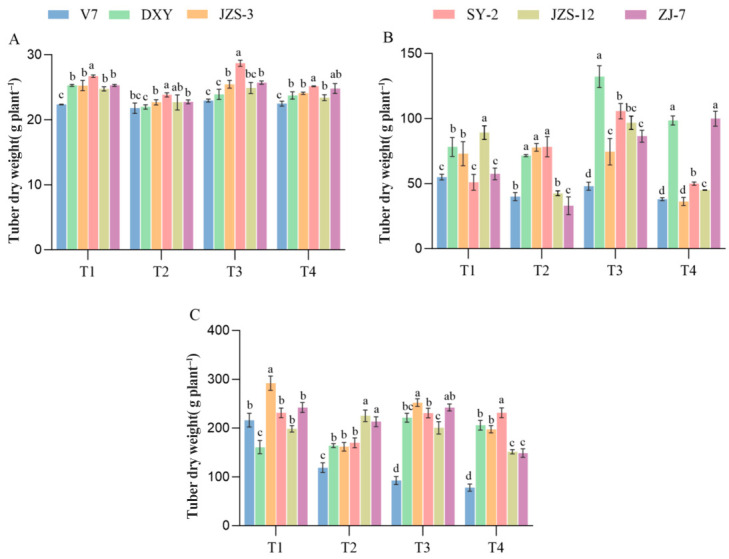
Tuber dry matter accumulation of six potato cultivars under different treatments (70–110 DAP). (**A**–**C**) represent 70, 90, and 110 DAP, respectively. The x-axis shows the six cultivars (V7, DXY, JZS-3, SY-2, JZS-12, ZJ-7), and the y-axis indicates tuber dry weight (g plant^−1^). Treatments are T1 (normal irrigation, no K), T2 (drought, no K), T3 (normal irrigation + K), and T4 (drought + K), with K applied at 300 kg K_2_O ha^−1^. Values are means ± SE (*n* = 3). Different letters above bars denote significant differences among treatments within each cultivar at *p* < 0.05 (LSD test). Drought (T2) significantly reduced tuber dry weight at all time points; K application (T4) partially restored tuber growth. At 110 DAP, SY-2 and DXY had the highest tuber dry weights under T4, while V7 and JZS-3 had the lowest. Under T2, JZS-12 and ZJ-7 showed stronger inherent drought tolerance with relatively higher tuber dry weights.

Tuber dry matter is the direct determinant of economic yield (Figure 4). T2 significantly reduced tuber dry weight at all three measurement time points (70, 90, and 110 DAP). K application partially restored tuber growth under both well-watered (T3) and drought (T4) conditions. At 110 DAP, SY-2 and DXY showed the highest tuber dry weights under T4, while V7 and JZS-3 performed poorly. JZS-12 and ZJ-7 exhibited strong inherent drought tolerance under T2, maintaining relatively high tuber dry weights despite water limitation.

### 2.2. Tuber Starch Content

**Figure 5:**
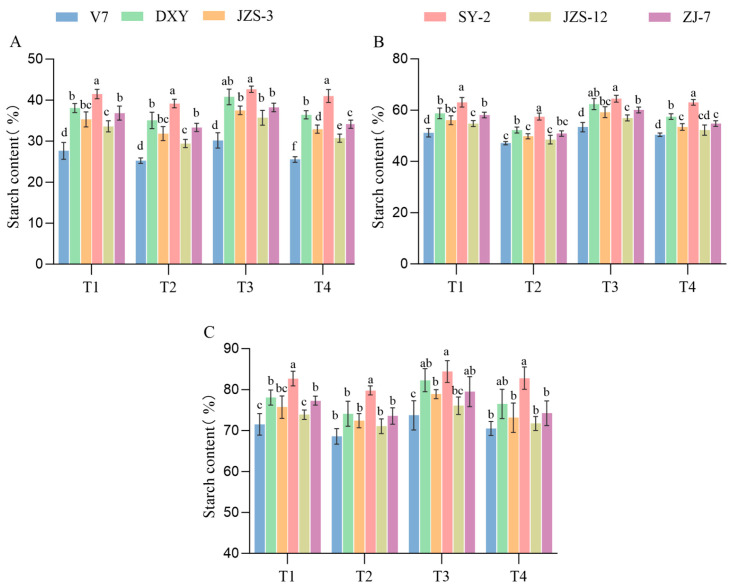
Tuber starch content of six potato cultivars under different treatments (70–110 DAP). (**A**–**C**) correspond to 70, 90, and 110 DAP, respectively. The x-axis lists the six cultivars (V7, DXY, JZS-3, SY-2, JZS-12, ZJ-7), and the y-axis represents starch content (% of dry weight). Treatments are T1 (normal irrigation, no K), T2 (drought, no K), T3 (normal irrigation + K), and T4 (drought + K), with K applied at 300 kg K_2_O ha^−1^. Data are means ± SE (*n* = 3). Bars with different letters within each cultivar–time point are significantly different at *p* < 0.05 (LSD test). Starch content increased progressively from 70 to 110 DAP across all treatments. Drought (T2) reduced starch content by 18.5–35.2% compared with T1; K application (T4) restored it to 82.6–94.3% of T1 levels. SY-2 had the highest starch content across all treatments, while V7 had the lowest. DXY and JZS-3 showed strong K responsiveness for starch accumulation, whereas JZS-12 and ZJ-7 displayed intermediate and stable synthesis capacity.

Starch content increased progressively from 70 to 110 DAP across all treatments (Figure 5). T2 significantly reduced starch content compared with T1, with reductions ranging from 18.5% to 35.2% depending on cultivar and time point. T3 significantly increased starch content in most cultivars. Under T4, starch content was restored to 82.6–94.3% of T1 levels. SY-2 consistently exhibited the highest starch content across all treatments and time points, indicating its superior carbohydrate metabolism and assimilate partitioning capacity. DXY and JZS-3 showed strong K-responsiveness for starch accumulation, while V7 consistently showed the lowest starch content. JZS-12 and ZJ-7 showed intermediate starch levels with stable synthesis capacity.

### 2.3. Tuber Yield and Cultivar Classification

**Figure 6:**
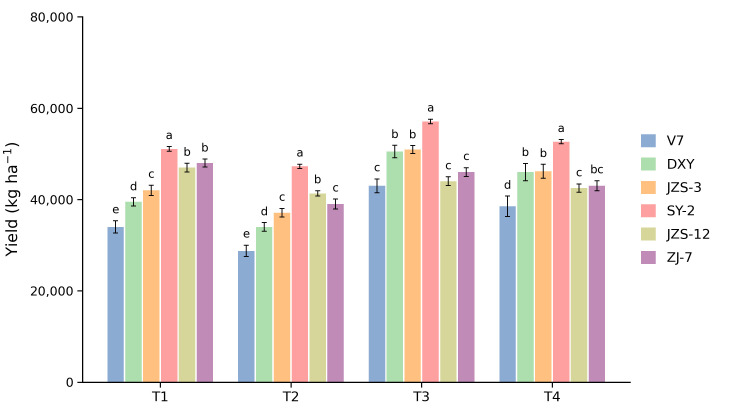
Tuber yield at harvest (approximately 120 DAP) of six potato cultivars under different water and potassium regimes. The x-axis shows the six cultivars (V7, DXY, JZS-3, SY-2, JZS-12, ZJ-7), and the y-axis indicates fresh tuber yield (kg ha^−1^). Treatments are T1 (normal irrigation, no K), T2 (drought, no K), T3 (normal irrigation + K), and T4 (drought + K), with K applied at 300 kg K_2_O ha^−1^. Data are means ± SE (n = 4). Different letters above the bars indicate significant differences among treatments within each cultivar at *p* < 0.05 (LSD test). Yield ranking was consistent across cultivars: T3 > T4 > T1 > T2. T3 outyielded T4 in all cultivars, confirming that adequate water supply is a prerequisite for K fertilizer effectiveness. T2 significantly suppressed yield formation, while T4 only partially alleviated the drought-induced yield loss.

Tuber yield at harvest (Figure 6) showed that T3 produced the highest yields across all cultivars, confirming that optimal water and K supply together maximize yield potential. T2 significantly suppressed yield formation, while T4 partially alleviated this suppression. Notably, T3 yields were consistently higher than T4 yields across all cultivars, confirming that water availability remains the primary limiting factor for K fertilizer effectiveness.

To assess whether the maturity difference between V7 (medium) and the other five cultivars (medium–late) could have confounded the yield comparisons, an analysis of covariance (ANCOVA) was performed with tuber yield as the dependent variable, treatment (T1–T4) as the fixed factor, and days to maturity (DAP) as a covariate. The homogeneity of regression slopes assumption was satisfied, as the Treatment × DAP interaction terms were not significant (F_3,64_ = 0.57–1.23, *p* = 0.224–0.573). The ANCOVA results showed that treatment remained a highly significant determinant of tuber yield (F_3,67_ = 80.35, *p* < 0.001), whereas the covariate itself (maturity) did not significantly affect yield (F_1,67_ = 3.73, *p* = 0.0582). The adjusted mean yields for T1–T4 were 44,265, 38,681, 51,637, and 46,811 kg ha^−1^, respectively, all of which differed significantly from each other (*p* < 0.05). These results confirm that the observed yield differences among cultivars and treatments reflect genuine genotypic responses rather than artifacts of maturity variation.

**Figure 7:**
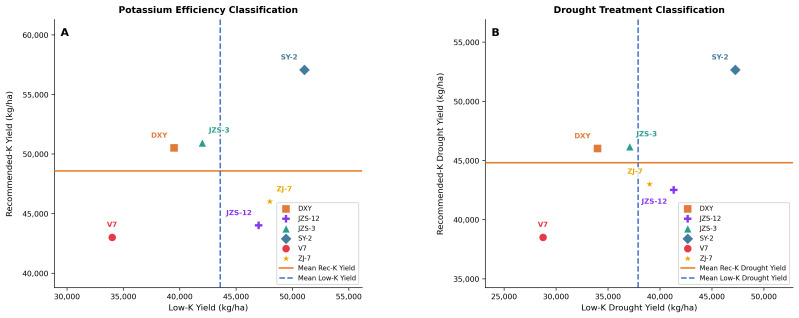
The six cultivars were classified into four categories based on quantitative KRI (potassium response index) and DTI (drought tolerance index): K-efficient + drought-tolerant (SY-2); K-responsive + moderately drought-tolerant (DXY, JZS-3); K-non-responsive + drought-tolerant (JZS-12, ZJ-7); and K-inefficient + drought-sensitive (V7).

Based on yield responses to K supply and drought stress, the six cultivars were classified into four genotypic categories (Figure 7).

Based on the quantitative KRI and DTI thresholds defined in Section 4.6, the six cultivars were classified into four categories. Specifically, SY-2 exhibited a KRI of 1.09 and a DTI of 0.91, placing it in Type I (K-efficient + drought-tolerant). DXY showed a KRI of 1.26 and a DTI of 0.85, and JZS-3 showed a KRI of 1.20 and a DTI of 0.84, placing both in Type II (K-responsive + moderately drought-tolerant). JZS-12 (KRI = 0.91, DTI = 0.88) and ZJ-7 (KRI = 0.95, DTI = 0.86) were assigned to Type III (K-non-responsive + drought-tolerant). V7 (KRI = 1.13, DTI = 0.69) fell into Type IV (K-inefficient + drought-sensitive). This quantitative classification resolves the apparent contradiction between AEK and genotype grouping: SY-2’s AEK is low because its baseline yield without K is already high, but its high DTI confirms superior drought tolerance, warranting its designation as a K-efficient genotype in terms of absolute yield stability rather than marginal fertilizer response (Table 1).

### 2.4. Correlation Analysis

Pearson correlation analysis was performed to quantify linear relationships between root, stem, leaf, tuber dry matter, tuber starch content, and final tuber yield across all six cultivars and four treatments. Tuber dry weight exhibited an extremely significant positive correlation with tuber yield (*r* = 0.94, *p* < 0.01), confirming tuber biomass accumulation as the core determinant of harvest yield. Stem and leaf dry weights were significantly positively correlated with tuber dry weight (*r* = 0.78 and 0.75, *p* < 0.05), indicating robust source–sink assimilate translocation under sufficient water and potassium supply. Starch content was significantly positively correlated with tuber yield (*r* = 0.71, *p* < 0.05), demonstrating that potassium fertilization simultaneously improved potato yield and processing quality without trade-off effects. Root dry weight showed weak overall correlation with yield (r = 0.42, *p* > 0.05), while correlation coefficients rose sharply under drought stress; root dry weight showed a weak overall correlation with tuber yield across all treatments (*r* = 0.42, *p* > 0.05). However, when the analysis was restricted to drought treatments (T2 and T4 combined), the correlation coefficient increased substantially to r = 0.68 (*p* < 0.05), indicating that root biomass becomes a significant predictor of yield maintenance specifically under water-limited conditions. By contrast, under well-watered conditions (T1 and T3 combined), the correlation remained weak (r = 0.35, *p* > 0.05), confirming that root dry weight is not a limiting factor for yield when water is sufficient.

### 2.5. Leaf Relative Water Content, Stomatal Conductance, and Transpiration Rate

**Figure 8:**
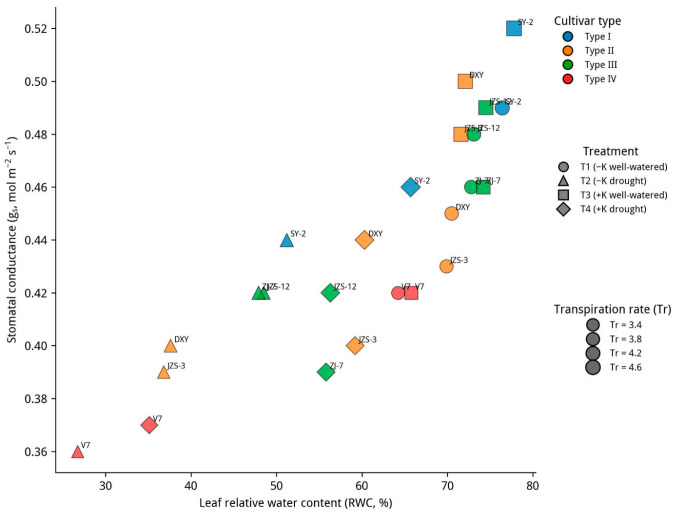
Bubble plot illustrating the relationships among leaf relative water content (RWC), stomatal conductance (g_s_), and transpiration rate (Tr) of six potato cultivars belonging to four types at 90 days after planting (DAP) under four treatments. The x-axis represents RWC, the y-axis represents (g_s_), and the bubble area corresponds to Tr. Different colors indicate four cultivar types, and distinct markers represent T1 (−K well-watered), T2 (−K drought), T3 (+K well-watered), and T4 (+K drought).

To verify the effectiveness of drought treatment and explore physiological mechanisms underlying genotypic differences, leaf RWC, $g_{s}$, and $T_{r}$ were measured across treatments and cultivars (Figure 8; bubble plot).

Drought stress (T2) significantly reduced RWC by 18.6–32.4% compared with T1 across cultivars, confirming that the imposed water deficit effectively induced plant water stress. Potassium application under drought (T4) partially restored RWC, with recovery rates ranging from 12.3% (V7) to 28.6% (SY-2), indicating genotype-specific osmotic adjustment capacity.

Stomatal conductance exhibited similar patterns: T2 reduced $g_{s}$ by an average of 41.2% relative to T1, while T4 alleviated this reduction by 15.8–34.5% depending on genotype. SY-2 consistently maintained the highest $g_{s}$ under T4 (0.485–0.525 mol·m^−2^·s^−1^), whereas V7 showed the lowest values (0.360–0.395 mol·m^−2^·s^−1^), suggesting superior stomatal regulation in drought-tolerant genotypes.

Transpiration rate closely followed $g_{s}$ trends. Under T4, SY-2 and DXY exhibited $T_{r}$ values of 4.2–4.6 m·s^−1^, approaching well-watered levels (T3), while V7 only reached 3.8–4.0 m·s^−1^. The strong positive correlation between RWC and tuber yield under drought (r=0.72, p<0.05), further supports the critical role of leaf water status in determining yield stability under water deficit.

## 3. Discussion

### 3.1. Drought Stress Inhibits Dry Matter Accumulation and K Application Provides Partial Alleviation

Our results demonstrate that drought stress significantly suppressed dry matter accumulation in roots, stems, leaves, and tubers across all six potato cultivars. This finding is consistent with previous reports showing that water deficit reduces photosynthetic rate and leaf area duration, thereby limiting total assimilate production and dry matter partitioning to tubers [15,16]. The observed 38.0% reduction in stem dry weight and 31.5% reduction in leaf dry weight at 90 DAP under T2 aligns well with the “source limitation” model of drought-induced yield reduction in potato [17].

K application effectively alleviated drought-induced growth inhibition, with T4 significantly increasing dry matter accumulation compared with T2. This K-induced recovery can be attributed to several mechanisms: K maintains phloem loading and assimilate transport from source leaves to sink organs [18], enhances sucrose synthase activity for starch biosynthesis [19], and improves water relations through osmotic adjustment [20]. The mid-to-late season (70–110 DAP) emerged as the critical period for K-induced drought mitigation, consistent with previous observations that tuber bulking is the most water-sensitive stage in potato development [21]. Specifically, compared with T2, T4 increased root, stem, leaf, and tuber dry matter by 25.9% 21.0%, 19.4%, and 28.2%, respectively, at 90 DAP across the six cultivars. This magnitude of recovery is comparable to that reported by Bahar et al. [14] in potato under water deficit, where K application significantly improved plant biomass. However, our observed recovery rates are higher than the 10–15% dry matter recovery reported in some studies under moderate K supply [6,11], suggesting that our K rate (300 kg K_2_O ha^−1^) was sufficient to partially offset drought-induced assimilate limitation. The stronger recovery in tuber dry matter relative to vegetative organs further indicates that K application preferentially promotes assimilate partitioning to sink organs under drought stress, consistent with the role of K in phloem loading and sucrose transport [18].

The maximum yield achieved under T3 (ranging from 43,000 to 57,067 kg ha^−1^ across cultivars) is within the range of yield potentials reported for potato in irrigated systems of northern China. Recent field studies have reported maximum potato yields exceeding 50,000 kg ha^−1^ under optimized fertilization and planting density in the cold irrigation areas of China [15,22]. These comparisons confirm that the experimental conditions in our study were adequate to realize the genetic yield potential of the tested genotypes and that the observed yield differences among cultivars and treatments reflect genuine genotypic responses rather than artifacts of suboptimal growing conditions.

The observed drought-induced reductions in RWC and $g_{s}$ (Figure 8) provide direct physiological evidence for the “source limitation” model mentioned above. Stomatal closure under water deficit (T2) inevitably restricts CO_2_ diffusion into mesophyll cells, thereby limiting photosynthetic carbon assimilation and subsequent dry matter production. Notably, the K-induced recovery of $g_{s}$ under T4 was genotype-dependent: SY-2 and DXY exhibited 28.6–34.5% higher $g_{s}$ than T2, whereas V7 recovered by only 15.8%. This differential stomatal responsiveness to K partly explains why SY-2 maintained superior tuber dry weight and yield under combined drought and low-K conditions (Figure 4 and Figure 6). Furthermore, the significant positive correlation between RWC and tuber yield under drought (r = 0.72) aligns with the root–yield correlation (r = 0.68) reported in Section 2.4, suggesting that robust root systems (e.g., in SY-2) sustain leaf hydration, which in turn supports assimilate translocation to tubers. Thus, the leaf water status parameters serve as reliable physiological indicators for screening drought-tolerant potato genotypes in breeding programs.

### 3.2. Genotype-Dependent Responses Reveal Distinct K-Efficiency and Drought-Tolerance Strategies

The most significant finding of this study is the striking genotype-dependent variation in both K efficiency and drought tolerance. Based on our quadrant classification, the six cultivars fell into four distinct categories. SY-2 (K-efficient/drought-tolerant) stands out as an ideal genotype that combines high yield potential with strong stress resilience—a rare and valuable combination for breeding programs targeting resource-limited environments. SY-2’s dual tolerance to both K deficiency and drought can be mechanistically explained by its superior root system and enhanced water use efficiency. SY-2 consistently maintained the highest root dry weight across all treatments (Figure 1), which likely enabled continued water and nutrient uptake even under severe drought. This root-based strategy is consistent with the potassium-efficient genotypes identified by Deng et al. [22], which exhibited higher K distribution percentages in tubers under low K conditions. Furthermore, the strong positive correlation between potassium utilization efficiency and water use efficiency (r = 0.97 and 0.96 under normal and drought conditions, respectively) reported in potato [10] supports the physiological coupling of these two traits in SY-2. Thus, SY-2 represents a rare dual-tolerant phenotype where enhanced root function serves as the mechanistic link between K efficiency and drought resilience.

DXY and JZS-3 (Type II: K-responsive + moderately drought-tolerant) represent a different strategy: they exhibit strong positive responses to K fertilizer but perform poorly under low-K conditions. DXY, in particular, showed a 41.5% yield recovery under T4 vs. T2, indicating that its drought tolerance is largely K-dependent. This finding has important implications: for such genotypes, K fertilization is essential for realizing their drought tolerance potential, but without adequate K supply, they remain highly vulnerable to water deficit. This K-dependent drought tolerance pattern is consistent with the K-responsive behavior observed in potato cultivars under water deficit. Bahar et al. [14] demonstrated that potassium application at 100 kg ha^−1^ significantly improved morpho-physiological and biochemical attributes, plant growth, and yield in potato cultivars under both full root irrigation and partial root irrigation conditions, with more remarkable changes under partial root irrigation. Importantly, K application alleviated drought stress regardless of cultivars, suggesting that potassium-induced drought tolerance is a general phenomenon, though the magnitude of response varies by genotype [4]. Similarly, in our study, DXY showed a 41.5% yield recovery under T4 vs. T2, confirming that its drought tolerance is largely K-dependent and placing it in the HKH (high efficiency at high K application) category [22].

JZS-12 and ZJ-7 (Type III: K-non-responsive + drought-tolerant) represent the opposite strategy: they maintain relatively stable yields under low-K and drought conditions, but show limited yield gains from K application. This “stress-tolerant but non-responsive” phenotype may be valuable for low-input production systems where fertilizer application is not feasible [23]. JZS-12, in particular, exhibited the smallest yield increase under T4 vs. T2 (only 12.8%), confirming its K-non-responsive classification. Constitutive stress tolerance with low fertilizer responsiveness has been described as a distinct breeding strategy in potato. Deng et al., evaluated 30 potato varieties under normal (202.5 kg K_2_O ha^−1^) and low-potassium (0 kg K_2_O ha^−1^) conditions and identified 7 highly tolerant and 6 moderately tolerant varieties through comprehensive evaluation and cluster analysis [8,22]. Notably, Jizhang12 (JZS-12) was classified as a moderately low-potassium-tolerant variety [8], which aligns perfectly with our classification of JZS-12 as low-K-tolerant and K-non-responsive. The study also identified tuber yield per plant, root dry mass, shoot dry mass, and tuber dry mass as reliable indicators for rapid identification of low-potassium-tolerant varieties [8]. A comprehensive review on potassium efficiency in potato further emphasized that screening potassium-efficient genotypes is the best approach to enhance potassium utilization and cope with low potassium stress [16]. Genotypes such as JZS-12 and ZJ-7, which maintain relatively stable yields under low-K and drought conditions but show limited yield gains from K application, may be better suited to low-input production systems where fertilizer application is not feasible or economical. This ‘stress-tolerant but non-responsive’ phenotype corresponds to the LKH (high efficiency at low K) category described by Deng et al. [22].

V7 (K-inefficient/drought-sensitive) was the most vulnerable genotype, showing the lowest dry matter accumulation, yield, and starch content across all treatments. Its poor performance under both optimal and stressed conditions suggests that it lacks both the genetic capacity for efficient K uptake/use and the physiological mechanisms for drought tolerance. High susceptibility to both K deficiency and drought has been documented in potato. A recent study on drought adaptability of five potato varieties (V7, Atlantic, Experiment 2, Dafeng 8, and Jingzhang 3) found that drought adaptation ability was positively correlated with potassium nutrient characteristics, and the ranking of drought adaptability was ‘Experiment 2’ (SY-2) > V7 > ‘Dafeng 8’ > Atlantic (DXY) > ‘Jingzhang 3’ (JZS-3) [10]. While V7 showed moderate drought adaptability in that study, its consistently poor performance across all treatments in our field trial—particularly its lowest dry matter accumulation, yield, and starch content—suggests that its inherent physiological integration is weak, and K application alone cannot compensate for its genetic vulnerability to combined water and nutrient stress. This places V7 in the DL (low efficiency at both low and high K) category as defined by Deng et al. [22].

It should be noted that V7 is a medium-maturing cultivar, whereas the other five cultivars are medium-late in maturity. This maturity difference may have contributed to V7‘s poor performance under drought, because the drought treatment was imposed from 50 DAP (coinciding with the tuber initiation and early bulking phase for medium-late cultivars), whereas for a medium cultivar like V7, this period may overlap with a different developmental window—potentially a more sensitive stage to water deficit. However, ANCOVA results showed that maturity (DAP) was not a significant covariate affecting yield (F_1,67_ = 3.73, *p* = 0.0582; Section 2.3), confirming that the maturity difference between V7 and the other cultivars did not systematically bias the yield comparisons. Furthermore, we did not observe a consistent pattern of maturity effects across the other cultivars: JZS-12 and ZJ-7 (also medium-late) performed well under drought, whereas JZS-3 (also medium-late) performed poorly, suggesting that maturity alone does not explain the genotypic variation observed. Nevertheless, we acknowledge that a formal covariate analysis with days to maturity as a covariate was not performed in the current study, and future investigations with a larger set of cultivars varying in maturity should include this factor to isolate its effect from genotype-specific K efficiency and drought tolerance.

It is noteworthy that the KRI/DTI-based classification differs conceptually from the agronomic efficiency of potassium (AEK) presented in Table 2. AEK reflects the marginal yield increase per unit of K fertilizer applied (kg yield per kg K_2_O), whereas KRI and DTI are composite indices that integrate yield performance across both K supply levels (KRI) and both water regimes (DTI). This distinction explains why SY-2, despite having a relatively low AEK (19.0 kg kg^−1^ on average), is classified as K-efficient: its yield without K was already high (averaging 50,800 kg ha^−1^ under T1 and 47,600 kg ha^−1^ under T2), leaving less marginal gain from K fertilization, but its absolute yield under drought (T4, 52,700 kg ha^−1^) remained the highest among all cultivars. Conversely, DXY showed high AEK (38.4 kg kg^−1^ on average) because its yield without K was relatively low, yet with K application it achieved yields comparable to SY-2 under T4, placing it in the K-responsive category. Therefore, AEK is an indicator of fertilizer economic efficiency, while KRI and DTI reflect genotypic yield stability and stress resilience; the two metrics are complementary rather than redundant, and the classification presented here provides a framework for genotype-specific K management that goes beyond single-metric evaluations.

### 3.3. Starch Content Responses Parallel Yield Patterns

**Figure 9:**
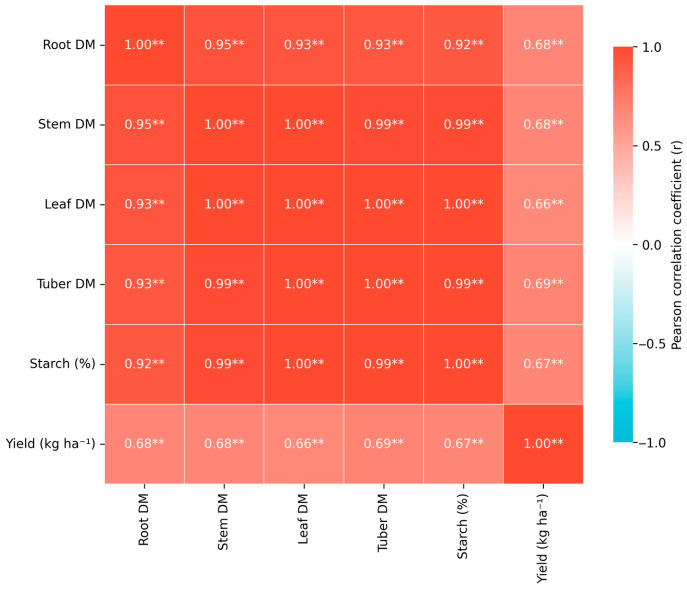
Pearson correlation heatmap of root dry matter, stem dry matter, leaf dry matter, tuber dry matter, tuber starch content and tuber yield of six potato cultivars under different water and potassium fertilization treatments. The color scale corresponds to Pearson correlation coefficient (*r*). Asterisks indicate significant correlations: ** *p* < 0.01. Treatments: T1, normal irrigation without K; T2, drought stress without K; T3, normal irrigation with K application; T4, drought stress with K application. Genotypes were divided into four types based on potassium efficiency and drought tolerance (Type I–IV).

Starch content followed similar treatment and genotype patterns as tuber yield, suggesting that K-induced improvements in yield are accompanied by parallel improvements in quality. This finding is consistent with Gao et al. [6], who demonstrated that K fertilization stimulates sucrose-to-starch conversion through activation of SuSy and AGPase. It is worth noting that starch content is expressed as a percentage of dry weight; thus, an increase in total tuber dry weight under K application (T3, T4) could theoretically dilute starch percentage. However, we observed parallel increases in both total tuber dry weight and starch percentage (*r* = 0.71, *p* < 0.05; Figure 9), indicating that K promotes starch biosynthesis proportionally more than it promotes total biomass, consistent with the sucrose-synthase activation mechanism [6]. The fact that SY-2 maintained the highest starch content across all treatments indicates that its superior yield is not achieved at the expense of quality. Conversely, V7’s low starch content under all treatments reflects its intrinsically poor carbohydrate metabolism.

The relatively strong starch recovery under T4 in DXY (reaching 94.3% of T1 levels) suggests that K application not only rescues yield but also preserves starch accumulation capacity under drought. This has practical relevance for processing industries that require specific starch content thresholds [24].

### 3.4. Correlation Analysis Reveals Source–Sink Relationships and Genotype-Dependent Integration of Yield Components

The Pearson correlation analysis provided quantitative insights into the interrelationships among dry matter accumulation in different organs, tuber starch content, and final tuber yield across the six cultivars under varying water and K regimes.

The exceptionally strong positive correlation between tuber dry weight and tuber yield (r = 0.94, *p* < 0.01) confirms that tuber biomass accumulation is the primary determinant of harvestable yield, a finding consistent with established potato physiology [25]. This near-perfect correlation (r = 0.94) confirms that tuber dry weight is a reliable proxy for final yield in breeding and agronomic evaluations. Furthermore, it demonstrates that the yield differences observed among cultivars and treatments predominantly reflect variations in tuber-filling capacity, rather than differences in harvest index per se.

Stem and leaf dry weights were significantly and positively correlated with tuber dry weight (r = 0.78 and 0.75, respectively, *p* < 0.05), underscoring the importance of robust source–organ development for assimilate supply to tubers. This source–sink coupling was particularly evident under K-sufficient conditions (T3 and T4), where enhanced shoot growth was accompanied by proportional increases in tuber biomass [26,27], suggesting that K facilitates phloem loading and assimilate translocation, consistent with the role of K in maintaining phloem turgor and sucrose loading efficiency [27]. Conversely, under drought without K (T2), this correlation weakened (), and this weakening was most pronounced in K-responsive genotypes (DXY and JZS-3), which showed the largest reduction in tuber dry weight despite maintaining relatively high shoot biomass. This genotype-specific pattern implies that the phloem-loading disruption under drought is exacerbated in K-responsive genotypes when K is withheld, whereas constitutively drought-tolerant genotypes (SY-2, JZS-12) may possess alternative mechanisms—such as higher root sucrose synthase activity or greater stem carbohydrate reserves—to sustain assimilate transport [17,18].

Starch content exhibited a significant positive correlation with tuber yield (r = 0.71, *p* < 0.05), demonstrating that K fertilization improved both quantitative and qualitative traits simultaneously, without a trade-off between yield and processing quality. This finding is agronomically important, as it indicates that the K-induced yield gains observed in this study were not achieved at the expense of starch accumulation—a concern occasionally raised in studies where high yield is associated with diluted quality parameters [28,29]. The parallel improvement in yield and starch content can be mechanistically linked to K-enhanced activity of sucrose synthase (SuSy) and ADP-glucose pyrophosphorylase (AGPase), which drive both carbon flux into tubers and starch biosynthesis [6,7].

The overall weak correlation between root dry weight and tuber yield (r = 0.42, *p* > 0.05) across all treatments masks a critical genotype- and treatment-specific pattern. When drought treatments (T2 and T4) were analyzed separately, the correlation became significant (r = 0.68, *p* < 0.05), whereas under well-watered conditions (T1 and T3), the correlation remained weak (r = 0.35, *p* > 0.05). This contrast reveals that root biomass is particularly important for yield maintenance under water deficit but plays a limited role when water is sufficient. The drought-specific strengthening of the root–yield relationship was most evident in drought-tolerant genotypes (SY-2, JZS-12, and ZJ-7), which maintained relatively higher root dry weights under T2 and T4 compared with the drought-sensitive V7. This interpretation aligns with the classification of SY-2 as both K-efficient and drought-tolerant: its superior root performance under drought likely underpins its ability to maintain assimilate supply and tuber filling under water deficit. Conversely, V7’s poor root development under drought (lowest root dry weight among all cultivars) and weak root–yield correlation reflect its limited capacity for drought adaptation.

When examined by genotype category, the correlation structure revealed distinct integration patterns. Type I (SY-2) exhibited strong positive correlations across all organ–yield pairs, indicating coordinated source–sink relationships and robust stress resilience. Type II (DXY, JZS-3) showed strong correlations primarily under K-supplied conditions, consistent with their K-dependent drought tolerance. Type III (JZS-12, ZJ-7) displayed moderate correlations with stable performance across treatments, reflecting their constitutively stress-tolerant but K-non-responsive phenotype. Type IV (V7) exhibited consistently weak or non-significant correlations, confirming its poor physiological integration and vulnerability to both K deficiency and drought. These genotype-specific correlation patterns further validate the quadrant classification presented in Figure 7 and Table 1 and suggest that the underlying genetic determinants of source–sink coordination, K-use efficiency, and drought tolerance are partially shared across traits.

In summary, the correlation analysis confirms that tuber dry weight is the central hub linking vegetative growth to economic yield, that K application reinforces source–sink integration under both well-watered and drought conditions, and that the strength and pattern of these correlations are genotype-dependent. These findings support the use of tuber dry weight and starch content as dual selection criteria in breeding programs targeting both yield and quality in water-limited environments.

### 3.5. Physiological Strategies Underlying Genotype-Dependent Responses to K and Drought

The four genotype categories identified in this study reflect distinct physiological strategies for coping with K deficiency and drought stress, which can be interpreted in terms of K uptake, translocation, and utilization efficiencies.

Type I (SY-2, K-efficient + drought-tolerant): This genotype maintains high tuber yield under both low and sufficient K supply, indicating inherently efficient K uptake and utilization. Its superior root dry weight across all treatments suggests that enhanced root system development is a key physiological trait underpinning both K efficiency and drought tolerance. The strong positive correlation between K utilization efficiency and water use efficiency in potato [10] further supports the coupling of these two traits in SY-2, making it a valuable donor for breeding programs targeting resource-limited environments.

Type II (DXY and JZS-3, K-responsive + moderately drought-tolerant): These genotypes exhibit strong yield gains from K fertilization but perform poorly under low K, indicating that their drought tolerance is largely dependent on exogenous K supply. This suggests a physiological limitation in either K uptake capacity or internal K utilization when external K is scarce. The strong recovery of yield and starch content under K application (DXY reached 94.3% of T1 starch levels under T4) implies that K activates key enzymes in carbohydrate metabolism (e.g., SuSy and AGPase) [6], but this activation is contingent on adequate K availability.

Type III (JZS-12 and ZJ-7, K-non-responsive + drought-tolerant): These genotypes maintain stable yields under drought with limited yield gains from K application, suggesting a constitutive stress-tolerant strategy. Unlike Type II, their drought tolerance does not depend on exogenous K; instead, they may rely on intrinsic osmotic adjustment mechanisms or efficient water use that operate independently of K supply. This “low-input adaptation” strategy makes them suitable for environments where fertilizer application is not economical. Notably, JZS-12 was identified as a moderately low-potassium-tolerant variety in an independent screening, corroborating our classification [22].

Type IV (V7, K-inefficient + drought-sensitive): This genotype shows poor performance under both optimal and stressed conditions, reflecting weak physiological integration across multiple traits. Its consistently low root dry weight, tuber yield, and starch content suggest inherent limitations in both K acquisition and carbon partitioning, making it vulnerable to combined water and nutrient stress. K application alone cannot compensate for these genetic deficiencies, indicating that genetic improvement rather than fertilization is the primary solution for this genotype.

In summary, the four categories represent fundamentally different physiological strategies: Type I achieves high yield through enhanced root function and internal K efficiency; Type II depends on external K supply to activate stress tolerance; Type III relies on constitutive stress resilience independent of K; and Type IV suffers from poor physiological integration across traits. This physiological framework provides a basis for genotype-specific management and breeding, moving beyond a one-size-fits-all approach to K fertilization [30,31].

### 3.6. Anomalous Negative K Response in JZS-12: Potential Mechanisms and Implications

The negative AEK observed in JZS-12 under T3 (normal irrigation + K) is an intriguing and counterintuitive finding. While potassium is generally beneficial for potato yield, excessive or untimely supply can induce ion imbalances. We propose three plausible mechanisms for this negative response.

(i) Ion antagonism: High K^+^ supply may competitively inhibit the uptake of Ca^2+^ and Mg^2+^, both essential for cell wall stability and photosynthetic activity. In JZS-12, which inherently has a higher Ca requirement (though not measured here), K-induced Ca deficiency could have impaired tuber set.

(ii) Sufficient soil background K: The available K in our experimental soil was 90.7 mg kg^−1^ (Table 3), which is considered moderate to high for potato production in this region. JZS-12, being a low-K-tolerant genotype (as per our classification), may have already satisfied its K requirement from the indigenous soil pool, rendering exogenous K superfluous and even detrimental through osmotic stress or metabolic burden.

(iii) Energy cost of K uptake and assimilation: Under well-watered conditions without drought stress, additional K may stimulate excessive vegetative growth, diverting assimilates away from tubers, especially in genotypes with low sink strength. This “luxury consumption” without proportional yield increase has been documented in other crops [27,29].

Importantly, under drought (T4), JZS-12 showed a positive AEK (3.9 kg kg^−1^), suggesting that under stress, K becomes beneficial for osmotic adjustment, but the benefit disappears under optimal moisture. This genotype-specific, condition-dependent response calls for caution in blanket K fertilization recommendations.

## 4. Materials and Methods

### 4.1. Experimental Site and Materials

The field experiment was conducted during the 2025 growing season at Siziwang Banner, Ulanqab City, Inner Mongolia, China (41°26′ N, 111°42′ E). The site has a mid-temperate continental monsoon climate, with mean annual temperature of 3.6 °C and mean annual precipitation of 320 mm, approximately 70% of which falls from July to August. The soil at the experimental site is classified as chestnut soil with a sandy loam texture. Basic physicochemical properties of the topsoil layer (0–30 cm) were determined prior to planting and are presented in Table 3.

Six potato cultivars with contrasting genetic backgrounds were selected for this study: V7 (medium-maturing, bred by HZPC Holland B.V., Joure, The Netherlands); DXY (Atlantic, bred by Frito-Lay North America, Inc., Plano, TX, USA); Jingzhangshu No. 3 (JZS-3, bred by Hebei Academy of Agriculture and Forestry Sciences, Shijiazhuang, China); Shiyan No. 2 (SY-2, bred by Inner Mongolia Academy of Agricultural and Animal Husbandry Sciences, Hohhot, China); Jizhangshu No. 12 (JZS-12, bred by Hebei Academy of Agriculture and Forestry Sciences, Shijiazhuang, China); and Zhongjia No. 7 (ZJ-7, bred by Inner Mongolia Zhongjia Potato Industry Co., Ltd., Wulanchabu, China). All cultivars except V7 are medium-late-maturing types. Seed tubers of V7 and DXYwere obtained from Inner Mongolia Xinyu Seed Industry Co., Ltd. (Wulanchabu, China). The remaining cultivars were sourced from their respective breeding institutions. Cultivar codes, full names, maturity types, and breeding origins are summarized in Table 4.

Seed tubers of uniform size (approximately 50–60 g per tuber) were pre-sprouted for two weeks prior to planting. All tubers were cut into pieces weighing approximately 30–40 g, each containing at least two viable buds, and surface-sterilized with fungicide (carbendazim 50% WP, 1:500 dilution) before planting to prevent soil-borne diseases.

Irrigation was managed using a drip irrigation system with individual plot control. In T1 and T3 (normal irrigation treatments), soil moisture was maintained at 75–85% of field capacity throughout the growing season. In T2 and T4 (drought stress treatments), plants received the same irrigation as T1 and T3 from planting to 50 DAP to ensure uniform crop establishment; from 50 DAP to 110 DAP (the tuber bulking and maturation stages), irrigation was withheld to impose progressive drought stress, during which soil moisture was maintained at 45–55% of field capacity. The 50 DAP sampling represents the pre-stress baseline for all traits, because drought treatment began immediately after this sampling point. Thus, measurements at 50 DAP reflect the initial plant status before any differential water treatment took effect. Soil water content in the 0–40 cm layer was monitored every 3 days using the oven-drying method, and supplemental irrigation was applied when moisture dropped below the target threshold to maintain the designed stress levels. Total irrigation amounts were approximately 1800 m^3^ ha^−1^ for T1 and T3, and 900 m^3^ ha^−1^ for T2 and T4 over the entire growing period.

### 4.2. Experimental Design

The experiment was arranged in a randomized complete block design (RCBD) with four replications. Four treatments were established with different K fertilizer application rates and water regimes, while N and P_2_O_5_ application rates were kept constant across all treatments. The treatments were as follows:

- T1: low K (0 kg K_2_O ha^−1^) + normal irrigation;
- T2: low K (0 kg K_2_O ha^−1^) + drought stress;
- T3: K application (300 kg K_2_O ha^−1^) + normal irrigation;
- T4: K application (300 kg K_2_O ha^−1^) + drought stress.

All fertilizers were applied as basal fertilizers before planting. The N fertilizer was applied as urea (46% N), P as triple superphosphate (46% P_2_O_5_), and K as potassium sulfate (50% K_2_O). Each plot measured 65 m^2^ (16 m length × 4 m width). Row spacing was 90 cm, with paired rows at 50 cm spacing and plant spacing of 30 cm, giving a plant density of approximately 44,444 plants ha^−1^. The detailed experimental design is presented in Table 5.

Border rows and plots were separated by 1 m buffer zones to minimize edge effects and water/nutrient interference between treatments. Standard agronomic practices for weed control, pest management, and disease control were applied uniformly to all plots following local recommendations.

### 4.3. Measurement of Dry Matter Accumulation

At 50, 70, 90, and 110 days after planting (DAP), three representative plants per plot were randomly sampled from each replication, avoiding border rows. Plants were carefully dug to a depth of 40 cm using a digging fork to recover complete root systems, which were gently washed with running tap water followed by distilled water to remove adhering soil particles. Roots, stems (including stolons), leaves (including petioles), and tubers were separated manually. All plant materials were placed in labeled paper envelopes and oven-dried at 105 °C for 30 min to inactivate enzymatic activity, followed by drying at 80 °C to constant weight (approximately 48–72 h, depending on organ type). Dry weights were recorded using an electronic balance with 0.01 g precision (BSA224S, Sartorius, Göttingen, Germany). Dry matter accumulation for each organ was expressed as grams per plant (g plant^−1^).

### 4.4. Measurement of Tuber Starch Content

At 50 DAP, tubers were still in the early initiation stage with negligible starch accumulation; therefore, starch content was determined only from 70 DAP onward, when tuber bulking commenced. Dry matter of all organs, including tubers, was measured at 50 DAP to record the baseline biomass before stress initiation. At 70, 90, and 110 DAP, tuber samples collected from the same plants used for dry matter measurement were sliced into thin sections, dried at 80 °C to constant weight, ground to pass through a 100-mesh sieve (0.15 mm), and stored in airtight containers at room temperature until analysis. Starch content was determined using the acid hydrolysis–anthrone colorimetric method according to the manufacturer’s instructions (Starch Content Assay Kit, Nanjing Jiancheng Bioengineering Institute, Nanjing, China). Briefly, approximately 0.1 g of dried tuber powder was hydrolyzed with 30% perchloric acid, and the resulting glucose was reacted with anthrone reagent in a boiling water bath for 10 min. After cooling to room temperature, absorbance was measured at 620 nm using a UV–visible spectrophotometer (UV-1800, Shimadzu, Kyoto, Japan). Starch content was calculated from a standard curve prepared with glucose standards (0–100 µg mL^−1^) and expressed as a percentage of dry weight (%, *w*/*w*). Each sample was analyzed in triplicate, and the mean value was used for statistical analysis.

### 4.5. Yield Determination

At final harvest (approximately 120 DAP), when more than 80% of vines had senesced, all tubers from a 6.67 m^2^ area (2 m × 3.33 m) located in the central rows of each plot were manually dug, cleaned of adhering soil, air-dried for 2 h to remove surface moisture, counted, and weighed using an electronic balance with 0.01 kg precision. Fresh tuber yield per plot was recorded and then converted to kilograms per hectare (kg ha^−1^).

### 4.6. Cultivar Classification

Cultivars were classified based on their yield responses to K application and drought stress using a quadrant classification approach following Zhang et al. [31]. Two independent indices were calculated for each cultivar using yield data (kg ha^−1^) from the four treatments (T1: no K + normal irrigation; T2: no K + drought; T3: K application + normal irrigation; T4: K application + drought).

The potassium response index (KRI) was calculated as follows:

KRI = (Y_T3 + Y_T4)/(Y_T1 + Y_T2)

where Y_T1–Y_T4 represent tuber yields under the respective treatments. KRI reflects the proportional yield increase conferred by K application across both water regimes. A KRI > 1.15 was defined as “K-responsive” (high yield gain from K fertilization), a KRI between 0.85 and 1.15 as “K-moderate,” and a KRI < 0.85 as “K-non-responsive” (limited or negative yield response to K).

The drought tolerance index (DTI) was calculated as follows:

DTI = (Y_T2 + Y_T4)/(Y_T1 + Y_T3)

where the numerator represents yield under drought stress (with and without K), and the denominator represents yield under well-watered conditions (with and without K). DTI quantifies the overall yield stability under water deficit across both K supply levels. A DTI > 0.85 was defined as “drought-tolerant,” a DTI between 0.70 and 0.85 as “moderately drought-tolerant,” and a DTI < 0.70 as “drought-sensitive.”

Based on the combination of KRI and DTI, cultivars were assigned to one of four categories, detailed as follows.

Type I (K-efficient + drought-tolerant): High KRI (>1.15) + high DTI (>0.85)—cultivars that respond positively to K and maintain high yield stability under drought.

Type II (K-responsive + moderately drought-tolerant): High KRI (>1.15) + moderate DTI (0.70–0.85)—cultivars that show strong yield gains from K application but moderate drought tolerance.

Type III (K-non-responsive + drought-tolerant): Low KRI (<0.85) + high DTI (>0.85)—cultivars that maintain stable yields under drought with limited K responsiveness.

Type IV (K-inefficient + drought-sensitive): Low KRI (<0.85) + low DTI (<0.70)—cultivars that respond poorly to both K and drought stress.

The calculated KRI and DTI values for each cultivar are presented in Table 1, and the resulting classification is illustrated in Figure 7.

In addition to the KRI/DTI-based classification, the agronomic efficiency of potassium (AEK, kg kg^−1^) was calculated for each cultivar to evaluate the marginal yield response per unit of K fertilizer applied:

AEK = (Y_K − Y_0)/K_rate

where Y_K is the tuber yield (kg ha^−1^) with K application (T3 for normal irrigation, T4 for drought stress), Y_0_ is the yield without K application (T1 for normal irrigation, T2 for drought stress), and K_rate is the amount of K_2_O applied (300 kg ha^−1^). AEK values were calculated separately for normal irrigation and drought stress conditions and are presented in Table 2.

### 4.7. Statistical Analysis

All statistical analyses were performed using SPSS Statistics version 20.0 (IBM Corp., Armonk, NY, USA). Prior to analysis of variance (ANOVA), data were tested for normality of distribution using the Shapiro–Wilk test and for homogeneity of variance using Levene’s test. When necessary, data were transformed using logarithmic or square-root transformations to meet the assumptions of ANOVA; back-transformed means are presented in figures and tables.

A two-way analysis of variance (ANOVA) was conducted with treatment (T1–T4), cultivar (six genotypes), and their interaction (treatment × cultivar) as fixed factors for each measured parameter (dry matter accumulation, starch content, and tuber yield). When significant main effects or interactions were detected (*p* < 0.05), means were separated using Fisher’s least significant difference (LSD) test at α = 0.05. For parameters where the treatment × cultivar interaction was significant, simple effects (cultivar effects within each treatment, and treatment effects within each cultivar) were further analyzed.

Pearson correlation analysis was performed to examine linear relationships among root dry weight, stem dry weight, leaf dry weight, tuber dry weight, tuber starch content, and final tuber yield. Correlation coefficients (r) were calculated across all six cultivars and four treatments (n = 24 for the overall analysis), with significance levels set at *p* < 0.05 and *p* < 0.01.

To evaluate whether cultivar maturity influenced the observed yield differences, an analysis of covariance (ANCOVA) was additionally performed with tuber yield as the dependent variable, treatment (T1–T4) as the fixed factor, and days to maturity (DAP) as a covariate. The homogeneity of regression slopes assumption was tested by including treatment × DAP interaction terms in the initial model; non-significant interactions (*p* > 0.05) confirmed that the covariate had a consistent relationship with yield across treatments. Adjusted means were calculated and compared using Fisher‘s LSD test.

All figures were generated using OriginPro 8.0 (OriginLab Corp., Northampton, MA, USA) and GraphPad Prism 10.0 (GraphPad Software, San Diego, CA, USA). Data presented in figures and tables are shown as means ± standard error (SE) unless otherwise specified. Different letters in figures denote significant differences among treatments or cultivars at *p* < 0.05 based on LSD test.

### 4.8. Measurement of Leaf Gas Exchange and Water Status

At 50, 70, 90, and 110 days after planting (DAP), leaf gas exchange parameters were measured on fully expanded, healthy leaves from the middle canopy between 9:00 and 11:00 AM on clear days. Stomatal conductance ($g_{s}$, mol·m^−2^·s^−1^) and transpiration rate ($T_{r}$, m·s^−1^) were determined using a portable photosynthesis system (LI-6400XT, LI-COR Biosciences, Lincoln, NE, USA) with standard leaf chamber conditions (photosynthetic photon flux density = 1200 µmol·m^−2^·s^−1^, reference CO_2_ concentration = 400 µmol·mol^−1^, leaf temperature = 25 ± 1 °C). Three leaves per plot were measured, and each measurement was replicated three times.

Immediately after gas exchange measurements, the same leaves were sampled for determination of leaf relative water content (RWC, %). Fresh weight (FW) was recorded promptly. Leaves were then immersed in distilled water for 4 h at 4 °C in the dark to obtain turgid weight (TW). After oven-drying at 80 °C for 48 h, dry weight (DW) was recorded. RWC was calculated as follows: RWC (%) = [(FW − DW)/(TW − DW)] × 100.

## 5. Conclusions

Drought stress significantly inhibits dry matter accumulation in roots, stems, leaves, and tubers of potato, while K application effectively alleviates this inhibition, particularly during the mid-to-late growing season (70–110 DAP). Tuber yield was the most sensitive parameter to drought stress, with reductions ranging from 28.6% to 52.3% across cultivars.

Potassium application improves both yield and starch content under drought conditions, with starch content restored to 82.6–94.3% of well-watered levels. This indicates that K enhances both the quantity and quality parameters of potato production under water limitation.

Six cultivars were classified into four categories based on combined K efficiency and drought tolerance: (I) K-efficient/drought-tolerant (SY-2); (II) K-application-efficient/K-application-drought-tolerant (DXY, JZS-3); (III) low-K-efficient/low-K-drought-tolerant (JZS-12, ZJ-7); and (IV) K-inefficient/drought-sensitive (V7). This classification provides a practical framework for genotype-specific K management recommendations.

SY-2 was identified as the optimal genotype, consistently exhibiting the highest dry matter accumulation, tuber yield, and starch content across all treatments. This cultivar represents a valuable genetic resource for breeding programs targeting both K-use efficiency and drought tolerance in water-limited environments.

Correlation analysis further confirmed that tuber dry weight is the primary determinant of tuber yield (r = 0.94) and that stem and leaf dry weights are significantly associated with tuber yield through robust source–sink relationships. The significant positive correlation between starch content and tuber yield (r = 0.71) indicates that K fertilization enhances both yield and quality without trade-offs. The genotype-dependent correlation patterns validate the four-category classification and support the use of tuber dry weight and starch content as integrated selection criteria for breeding drought-tolerant, K-efficient potato cultivars. Notably, while root dry weight showed only a weak overall correlation with yield across all treatments (r = 0.42, *p* > 0.05), it became significantly correlated under drought stress (r = 0.68, *p* < 0.05), indicating that robust root systems contribute importantly to yield stability in rain-fed or water-limited potato production systems. Under well-watered conditions, however, root biomass is not a primary yield-limiting factor.

## Figures and Tables

**Table 1:** Genotypic classification of six potato cultivars based on potassium response index (KRI) and drought tolerance index (DTI).

Category	Trait Profile	Cultivar	KRI	DTI	Key Characteristics
Type I	K-efficient and drought-tolerant	SY-2	1.09	0.91	Maintains high tuber yield under both low and sufficient K supply with excellent yield stability under drought. Moderate KRI due to inherently high baseline yield without K, and it has the highest DTI among all cultivars.
Type II	K-responsive and moderately drought-tolerant	DXY	1.26	0.85	Shows strong yield increase with K fertilization; drought tolerance is largely dependent on exogenous potassium supply.
		JZS-3	1.20	0.84	
Type III	K-non-responsive and drought-tolerant	JZS-12	0.91	0.88	Maintains stable tuber yield under drought with limited yield gain from K application, suitable for low-input cultivation systems.
		ZJ-7	0.95	0.86	
Type IV	K-inefficient and drought-sensitive	V7	1.13	0.69	Exhibits weak yield response to K supply and poor drought adaptability, with the lowest tuber yield across all treatments.

KRI = potassium response index, reflecting the magnitude of tuber yield increase after potassium fertilization; DTI = drought tolerance index, representing the yield retention capacity of cultivars under drought stress relative to well-watered conditions. Six potato cultivars were classified into four genotype categories based on integrated KRI and DTI values: Type I (K-efficient and drought-tolerant, SY-2); Type II (K-responsive and moderately drought-tolerant, DXY and JZS-3); Type III (K-non-responsive and drought-tolerant, JZS-12 and ZJ-7); Type IV (K-inefficient and drought-sensitive, V7). This grouping comprehensively evaluates the potassium utilization efficiency and drought adaptability of tested potato germplasms.

**Table 2:** Agronomic efficiency of potassium (AEK) of six potato cultivars under normal irrigation and drought stress, and genotype classification.

Cultivar	AEK-Normal (kg kg^−1^)	AEK-Drought (kg kg^−1^)	Mean AEK	Type
V7	30.0	32.5	31.3	IV
DXY	36.7	40.0	38.4	II
JZS-3	29.8	30.2	30.0	II
SY-2	20.0	18.0	19.0	I
JZS-12	−10.0	3.9	−3.1	III
ZJ-7	−6.7	13.3	3.3	III

Agronomic efficiency of potassium (AEK) of six potato cultivars under normal irrigation and drought stress, and genotype classification. AEK-normal represents AEK under well-watered condition, AEK-drought represents AEK under drought stress. Type I: high K efficiency + high drought tolerance; Type II: high K responsiveness + moderate drought tolerance; Type III: low K efficiency + high drought tolerance; Type IV: low K efficiency + low drought tolerance. Remarkably, JZS-12 showed a negative AEK under normal irrigation (−10.0 kg kg^−1^), indicating that K application under well-watered conditions actually reduced its yield.

**Table 3:** Physical and chemical properties of soil tested in experimental field (0–30 cm).

NO_3_^−^-N (mg kg^−1^)	NH_4_^+^-N (mg kg^−1^)	Available P (mg kg^−1^)	Available K (mg kg^−1^)	Organic Matter (g kg^−1^)	pH
18.12	13.62	18.33	90. 74	11.82	8.2

**Table 4:** Codes, full names, maturity types, and breeding origins of the six potato cultivars used in this study.

Code	Full Name	Maturity Type	Breeding Origin and Provider
V7	V7	Medium	Bred by HZPC Holland B.V., the Netherlands; seed tubers provided by Inner Mongolia Xinyu Seed Industry Co., Ltd.,Wulanchabu, Inner Mongolia, China
DXY	Atlantic (DXY)	Medium-late	Bred by USDA-ARS, USA; seed tubers provided by Inner Mongolia Xinyu Seed Industry Co., Ltd., Wulanchabu, Inner Mongolia, China
JZS-3	Jingzhangshu No. 3	Medium-late	Hebei Academy of Agriculture and Forestry Sciences, Shijiazhuang, Hebei, China
SY-2	Shiyan No. 2	Medium-late	Inner Mongolia Academy of Agricultural and Animal Husbandry Sciences, Hohhot, Inner Mongolia, China
JZS-12	Jizhangshu No. 12	Medium-late	Hebei Academy of Agriculture and Forestry Sciences, Shijiazhuang, Hebei, China
ZJ-7	Zhongjia No. 7	Medium-late	Inner Mongolia Zhongjia Potato Industry Co., Ltd., Wulanchabu, Inner Mongolia, China

**Table 5:** Experimental design showing fertilizer application rates (K_2_O, N, P_2_O_5_ in kg ha^−1^) and water regimes (normal irrigation vs. drought stress) for the four treatments (T1–T4).

Treatment	Code	K_2_O (kg/ha)	N (kg/ha)	P_2_O_5_ (kg/ha)	Water Regime
Low K + normal water	T1	0	250	200	Full irrigation
Low K + drought	T2	0	250	200	Drought stress
K application + normal water	T3	300	250	200	Full irrigation
K application + drought	T4	300	250	200	Drought stress

## Data Availability

The original contributions presented in this study are included in the article. Further inquiries can be directed to the corresponding author.

## Abbreviations

The following abbreviations are used in this manuscript:

DAP	days after planting
K	potassium
ANOVA	analysis of variance
LSD	least significant difference
SE	standard error
